# *Escherichia coli* bacteriuria in pregnant women in Ghana: antibiotic resistance patterns and virulence factors

**DOI:** 10.1186/s13104-018-3989-y

**Published:** 2018-12-17

**Authors:** Akua Obeng Forson, Wilson Bright Tsidi, David Nana-Adjei, Marjorie Ntiwaa Quarchie, Noah Obeng-Nkuramah

**Affiliations:** Department of Medical Laboratory Science, School of Biomedical and Allied Health Sciences, College of Health Sciences, University of Ghana, Legon, Accra, Ghana

**Keywords:** *Escherichia coli*, Bacteriuria, Pregnant women, Ghana

## Abstract

**Objectives:**

The relevance of *Escherichia coli* associated bacteriuria infection in pregnant women is poorly understood, despite these strains sharing a similar virulence profile with other pathogenic *E. coli* causing severe obstetric and neonatal infections. We characterized and determined the antimicrobial susceptibility, resistance genes and virulence profiles of 82 *E. coli* isolates associated with asymptomatic bacteriuria in some pregnant in Ghana from February to August 2016 using Kirby–Bauer disc diffusion and polymerase chain reaction.

**Results:**

High levels of antimicrobial resistance were observed to ampicillin (79.3%), tetracycline (70.7%) and cotrimoxazole (59.8%), except for cefuroxime (32.9%). Resistance genes analyses revealed 58.5% were positive for *Bla*_TEM_ and 7.3% for *aph(3)*-*Ia*(aphA2). Virulence factors (VFs) was more widespread in pregnant women in the 2nd and 3rd trimesters than 1st trimester. VFs relating to adhesion (*pap*C and *iha*), Protectins (*tra*T), aerobactin acquisition (*iut*A) and iron acquisition systems (*fyu*A and *irp*2) were more prevalent in the resistant *E. coli* isolates. This study provides evidence for a link in bacteriuria and transmission of extra-intestinal *E. coli* in pregnant women to cause multi-resistant obstetric or neonatal infections. Considering the involvement of extra-intestinal *E. coli* in infections, results are helpful to develop strategies to prevent maternal and/ neonatal infections.

**Electronic supplementary material:**

The online version of this article (10.1186/s13104-018-3989-y) contains supplementary material, which is available to authorized users.

## Introduction

In pregnant women, the odds of acquiring urinary tract infections (UTI) from untreated bacteriuria is high, with consequent risk for preterm labour [1]. Pregnant woman diagnosed with bacteriuria are thus offered antibiotics to prevent complications [2, 3]. The extraintestinal pathogenic *E. coli* (ExPEC) are a major cause of UTI in pregnancy [4–6]. The ExPEC harbour diverse but specific virulence factors (VFs) with the potential to colonize highly specialized ecological niches, such as the urogenital tract [7–11]. Furthermore, multidrug-resistance traits in many ExPEC strains involved in bacteriuria is increasing accounting for considerable amount of morbidity, and can lead to significant mortality in pregnant women with UTI [12–15]. There is however paucity of data in sub-Sahara Africa where such infections are likely to be common and devastating due to high exposure to infectious organisms and limited access to health care services [1, 13–20]. In Ghana, the aetiology of *E. coli* in bacteriuria are well documented [21–25] but often limited to phenotypic tests with little knowledge on virulence factors. This study aimed to characterize ExPEC strains, and determine their virulence and antimicrobial resistance potential in urine samples of pregnant women.

## Main text

### Materials and methods

#### Sample collection and processing

The approximated sample size of 400 was calculated using the formula N = Z^2^P (1 − P)/D^2^ where; N = sample size; Z = 95% (1.96) confidence interval; P = previous reported prevalence of UTI among pregnant women in Ghana (56.5%) [26], and D = allowable margin error of 0.05. The 400 pregnant women were recruited into this study after the provision of informed consent. Pregnant women on antibiotics were excluded. A self-administered questionnaire was used to obtain information on demographic and socio-economic characteristics (Additional file 1: S1 file). Mid-stream-clean-catch urine from participants were inoculated onto cysteine lactose electrolyte deficient (CLED) agar and incubated at 37 °C for 24 h [27]. Bacteria isolates were speciated with API 20E identification system (bioMerieux, France). For purposes of this study, only *E. coli* cultures were further analyzed [28].

#### Antimicrobial susceptibility

*Escherichia coli* cultures were subjected to Kirby–Bauer method of sensitivity testing per guidelines of the Clinical and Laboratory Standard Institute (CLSI) [29]. The following antibiotics were used: ampicillin (10 μg), tetracycline (30 μg), cotrimoxazole (25 μg), nalidixic acid (30 μg), nitrofurantoin (300 μg), gentamicin (10 μg) and cefuroxime (30 μg). These antibiotics were selected because they are commonly used antibiotics for the treatment of bacterial infections in the general populace [21, 25]. Control strains included *E. faecalis* ATCC 29212, *E. coli* ATCC 25922, and *Staphylococcus aureus* ATCC 29213 (Additional file 1: S2 file).

#### Molecular characterization

Colonies of fresh bacterial culture were suspended in 200 ml of sterile water. The suspension was heated at 98 °C for 10 min and centrifuged at 17,900*g* for 5 min. The supernatant was recovered and used as templates for polymerase chain reactions (PCR). Gene amplification was done for *bla*_TEM_; aminoglycoside resistant genes [*Ia*(aphA1), *Ia*(aphA2)], integrase genes I with II; and 18 virulent factors for ExPEC adhesions, toxins, iron capture systems, protectins, uropathogenic specific protein and aerobactin system (Additional file 1: S3 file) as previously reported [11, 30, 31]. Virulence factors (Vfs) for all the *E. coli* isolates were tested using primers by Johnson et al. [5, 6, 9, 11, 30] and other authors [31–39] as these were reported to be sufficient to identify ExPECs. The virulent genes were amplified in 6 primer pools [1 (*iron*, *sfa*, *iut*A, *hra*), 2 (*pap*A, *KpsM*TIII, *ire*A, *ibe*A), 3 (*pap*G1, *pap*GII, III, *iha*, *omp*T, *KpsM*TII), 4 (*iuC*, *Cnf*1, *irp*2), 5 (*hly*D, *usp*, *tra*T), and 6 (*pap*C, *sat*, *Fyu*A)] [11, 30, 31].

#### Statistical analysis

Data was analysed using GraphPad Prism software, version 6. Bacteriuria was defined as bacterial growth > 10^5^ colony forming units/mL per urine sample on CLED. Associations between socio-demographic characteristics and development of UTI, phenotypic resistance, and virulence factors were done using Chi square test. p-values < 0.05 were considered significant.

### Results

**Fig. 1:**
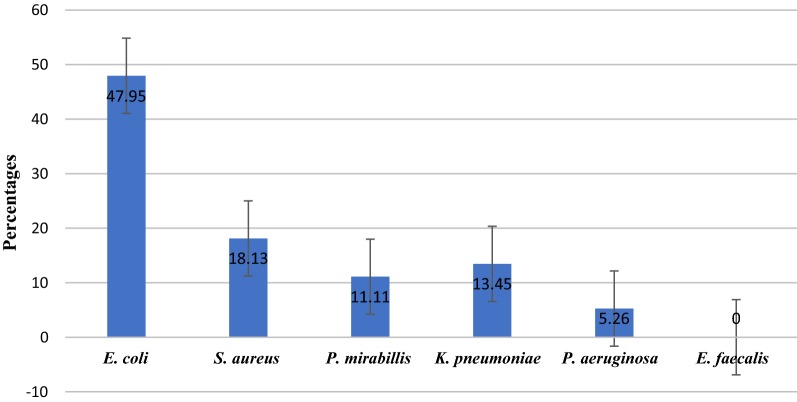
Distribution of isolated bacteria

Overall, 42.8% (n = 171) of 400 participants had bacteriuria. *Escherichia coli* was the most predominant isolate (47.95%), followed by *Staphylococcus aureus* (18.1%), and *Klebsiella pneumoniae* (13.45%) (Fig. 1).

Forty-four percent of pregnant women aged 13 to 19 years had UTI, with *E. coli* accounting for 33.3%. Out of the 228 patients (aged 20–29 years), 96 (42.11%) had UTI with 52.08% associated with *E. coli* (Additional file 1: S4 file). The rate of UTI in the 30–39 age groups was 42.18% and *E. coli* was associated with 50%. The differences in the rate of UTI among the various age groups was statistically non-significant (p = 0.706). Basic level education (62.7%) and secondary education (25.5%) were the common levels of education (Additional file 1: S4 file). One hundred and ten (43.8%) of the women with basic education had UTI and 45.5% were infected with *E. coli*. Forty-five percent of the pregnant women with secondary education had UTI and *E. coli* was associated with 52.2% [23]. The differences in rate of UTI in relation to educational status was statistically non-significant (p = 0.262). Significant bacteria growth of 42.3% and 49.1% was found for women in 2nd and 3rd trimesters (Additional file 1: S4 file). Fifty-five percent of first trimester pregnant women with UTI were associated with *E. coli.* Chi square exact test revealed an association with gestational age and the development of UTI (p = 0.002).

*Escherichia coli* isolates were highly resistant to ampicillin (79.3%) and tetracycline (70.7%) (Table 1). Whilst a resistance of 59.8% and 48.8% were found for cotrimoxazole and nalidixi acid, the least resistance was to cefuroxime (32.9%) and nitrofurantoin (35.4%).

**Table 1:** Antibiotic resistance pattern of *E. coli* isolate

Antibiotic	Hospitals (no.)					Total (n = 82, %)
	St. Joseph hospital (n = 23, %)	Volta regional hospital (n = 11, %)	Mary Theresa hospital (n = 16, %)	Ketu South Mun. hospital (n = 18, %)	St. Anthony hospital (n = 14, %)	
Ampicillin	20	8	11	15	11	65 (79.3)
Tetracycline	18	8	10	12	10	58 (70.7)
Cotrimoxazole	16	6	8	9	10	49 (59.8)
Nalidixic acid	8	6	8	11	7	40 (48.8)
Nitrofurantoin	7	4	5	9	4	29 (35.4)
Gentamicin	6	4	6	10	8	34 (41.5)
Cefuroxime	6	3	3	12	3	27 (32.9)

In total, 49 (59.7%) ampicillin resistant isolates contained *Bla*_TEM_ (Table 2). Pregnant women in the 2nd (24 isolates) and 3rd (18 isolates) trimesters had *E. coli* isolates with more *Bla*_TEM_ gene compared to women in their 1st trimesters (5 isolates). The aminoglycoside genes *aph(3)*-*Ia*(aphA2) for gentamicin resistance was in 6 isolates from pregnant women in their 2nd and 3rd trimesters (Table 2). All the *E. coli* isolates were screened for the presence of *int*I and *int*II, however only 10 of isolates were positive for *int*I, whilst two *E. coli* isolates contained *int*II, 58 of the isolates did not possess either *int*I or *int*II.

**Table 2:** Distribution of VFs and resistance genes in *E. coli* isolates

Age	1st trimester		2nd trimester		3rd trimester	
	Resist. genes	Tested Vfs (no. of positives)	Resist. genes	Tested Vfs (no. of positives)	Resist. genes	Tested Vfs (no. of positives)
13–19	*Bla*_TEM_	*iut*A, *irp*, *pap*C, *Fyu*A(1)	*Bla*_TEM_, *int*1	*irp*, *tra*T, *Fyu*A(1)	*Bla*_TEM_, int1, *aphA*2	*iut*A, *irp*, *pap*C, *Fyu*A(1)
			*Bla*_TEM_	*iut*A, *iha*, *irp*, *tra*T(1)	*Bla*_TEM_	*iut*A, *iron*, *iha*, *irp*, *tra*T, *pap*C, *Fyu*A(1)
			*int*1	*iut*A, *pap*A, *iha*, *irp*, *tra*T, *pap*C, *Fyu*A(1)	*Bla*_TEM_	*iron*, *ire*, *pap*A, *iha*, *irp*, *pap*C, *Fyu*A(1)
20–29	–	*irp*, *tra*T(1)	*Bla*_TEM_	*iha*, *irp*, *tra*T(1)	–	*iha*, *irp*(1)
	*Bla*_TEM_	*iut*A, *iron*, ire, papG1(1)	*Bla*_TEM_, *int*1	*irp*(1)	*Bla*_TEM_	-(2)
	*int*1	*iut*A, *pap*A, *iha*, *irp*, *tra*T(1),	*Bla*_TEM_, *int*1	*tra*T(1)	*Bla*_TEM_	*iut*A, *hra*, *iha*, *irp*, *Fyu*A(1)
	*Bla*_TEM_, *int*1	*iut*A, *omp*T, *pap*G1, *irp*, *tra*T(1)	–	*iha*, *omp*T(1)	*Bla*_TEM_	*iut*A, *pap*G1, *irp*, *tra*T, *Fyu*A(2)
	*int*1	*iut*A, *sfa*, *hra*, *iron*, *iha*, *omp*T, *irp*2, *tra*T(1)	*Bla*_TEM_	*irp*, *tra*T, *Fyu*A(1)	*Bla*_TEM_	*iut*A, *omp*T, *irp*, *pap*C, *Fyu*A(1)
	*int*2	*iut*A, *sfa*, *iron*, *iha*, *kpsMT*II(1)	*Bla*_TEM_	*iut*A, *tra*T, FyuA(1)	*Bla*_TEM_, *int*1	*iut*A, *irp*, *tra*T, *pap*C, *Fyu*A(1)
		*irp*, *tra*T, *pap*C, *Fyu*A(1)				
	*int*2	*iut*A, *sfa*, *pap*A, *iron*, *pap*A	*Bla*_TEM_	*iha*, *irp*, *tra*T(1)	*Bla*_TEM_	*iron*, *irp*, *tr*aT, *pap*C, *Fyu*A(1)
		*Iha*, *irp*, *pap*C, *Fyu*A(1)	*Bla*_TEM_, *int*1	*iut*A, *irp*, *iu*C(1)	*Bla*_TEM_, *aphA*2	iutA, *omp*T, *irp*, *tra*T, *Fyu*A(1)
			–	*iut*A, *omp*T, *irp*, *tra*T(1)	*Bla*_TEM_	*iut*A, *omp*T, *irp*, *iuc*, *pap*C, *Fyu*A(1)
			–	*iut*A, *hra*, *ire*, *tra*T(1)	*Bla*_TEM_	*iut*A, *iron*, *omp*T, *irp*, *pap*C, FyuA(1)
			*Bla*_TEM_, *aphA*2	kpsMTIII, *irp*, *pap*C, *Fyu*A(1)	–	*iut*A, *pap*A, *iron*, *irp*, *pap*C, *Fyu*A(1)
			*Bla*_TEM_	*iut*A, *iha*, *irp*, *Fyu*A(1)	–	*iut*A, *sfa*, *pap*A, *iha*, *irp*, *pap*C, *Fyu*A(1)
			*Bla*_TEM_	*omp*T, *irp*, *tra*T, *usp*(1)	–	*sfa*, *hra*, *iha*, *kpsM*TII, *irp*, *tra*T, *Fyu*A(1)
			*Bla*_TEM_	*iha*, *irp*, *tra*T, *pap*C, *Fyu*A(1)		
			–	*iut*A, *irp*, *tra*T, *pap*C, *Fyu*A(1)		
			*Bla*_TEM_	*iha*, *pap*G1, *irp*, *pap*C, *Fyu*A(1)		
			*Bla*_TEM_, *aphA*2	*iut*A, *iron*, *ire*, *iha*, *omp*T, *tra*T, *pap*C, *Fyu*A(1)		
			*Bla*_TEM_, *aphA*2	*iut*A, *iron*, *ire*, *iha*, *omp*T, *tra*T, *pap*C, *Fyu*A(1)		
			*Bla*_TEM_	*sfa*, *iron*, *ire*, *iha*, *irp*, *tra*T, *hly*D, *pap*C, *Fyu*A(1)		
			0	*iut*A, *sfa*, *iron*, *kpsMT*III, *iha*, *omp*T, *tra*T, *pap*C, *Fyu*A(1)		
			*Bla*_TEM_	*sfa*, *iron*, *ire*, *pap*A, *iha*, *irp*, *tra*T, *hly*D, *pap*C, F*yuA*(1)		
30–39	*Bla*_TEM_, *int*1	*irp*, *tra*T, *Fyu*A(1)	*Bla*_TEM_	*irp*(1)	0	*iut*A, *irp*, *pap*C, *Fyu*A(1)
	*Bla*_TEM_	*iut*A, *omp*T, *irp*, *pap*C, *Fyu*A(1)	–	*iut*A, *iha*(1)	0	*iha*, *irp*, *tra*T, *usp, Fyu*A(1)
			*Bla*_TEM_	*iut*A, *iron*, *iha*(1)	*Bla*_TEM_, *int*1	*iut*A, *pap*G1, *irp*, *pap*C, *Fyu*A(1)
			*Bla*_TEM_	*iut*A, *iha*, *irp*, *tra*T(2)	–	papA, *irp*, *tra*T, *pap*C, *Fyu*A(1)
			–	*iut*A, *omp*T, *irp*, *Fyu*A(1)	*Bla*_TEM_	*iut*A, *ibe*, *omp*T, *kpsM*TII, *tra*T, *Fyu*A(1)
			*Bla*_TEM_	*iut*A, *irp*, *iu*C, *pap*C, *Fyu*A(1)	*Bla*_TEM_, *int*1	*iut*A, *omp*T, *irp*, *tra*T, *pap*C, *Fyu*A(1)
			*Bla*_TEM_	*iut*A, *ire*, *omp*T, *irp*, *tra*T(1)	*Bla*_TEM_, *aphA*2	*iu*tA, *pap*A, *omp*T, *irp*, *tra*T, *Fyu*A(1)
					–	*iut*A, *pap*A, *iha*, *irp*, *tr*aT, *pap*C, *Fyu*A(1)
40–49	–	–	–	*irp*, *Fyu*A(1)	*Bla*_TEM_	*iut*A, *pap*A, *irp*, *tra*T, *pap*C, *Fyu*A(1)
					*Bla*_TEM_	*ire*, *pap*A, *iha*, *irp*, *tra*T, *pap*C, FyuA(1)
Total no. genes/ExPECs	9	12	25	30	18	25

The distribution of the 82 *E. coli* isolates in relation to virulence genes from the various groups of pregnant women revealed 75.6% (62 isolates) *E. coli* contained two or more virulence genes (VFs) (Table 2). The virulence score used to classify the ExPEC isolates was calculated using the total number of VFs genes. Isolates were classified as ExPEC if they were positive for two or more of the tested virulence genes [5]. The *iut*A (aerobactin acquisition), *pap*C and *iha* (adhesins), *fyu*A and *irp*2 (iron capture systems), *tra*T (protectins) were the common detected genes, whereas *usp* (uropathogenic-specific proteins) and some of the adhesin genes (*hra*, *ibe*A, and *pap*G1) were the least.

VFs was widespread in pregnant women in the 2nd (30 isolates) and 3rd (25 isolates) trimesters than 1st trimester (12 isolates) (Table 2). In this study, all the *E. coli* isolates in women in their 1st trimester were ExPEC. Whilst 19 pregnant women in their 2nd trimesters (20–29 years) were positive for ExPEC, 13 women in the 3rd trimester (20–29 years) were positive. In addition, in the age group 40–49 years, only 2 women in the 3rd trimester were ExPEC positive, whilst one woman in the 2nd trimester was positive (Table 2).

### Discussion

There are few studies on the antimicrobial susceptibility and/or virulence of *E. coli* isolates colonizing the genital tract of pregnant women [39–41]. However, no studies have been carried out to compare virulence factors and antimicrobial resistance in *E. coli* from pregnant in Ghana. This study revealed the 42.75% of the pregnant women with UTI was slightly lower than the 56.5% previously reported in Ghana by Boye et al. [26]. Although findings are similar to 47.5% from Nigeria [42], it is lower than the 85% reported by Turay et al. [43]. However, the 42.75% in this study is higher than reports from Thailand (5.1%) and Ethiopia (18.8%) [44, 45]. The difference in prevalences may be attributed to varied genital hygiene and socioeconomic conditions [46]. *E. coli* accounted for 47.95% of the UTI cases in the pregnant women. This is in conformity with previous studies from Sudan, Bangladesh, and Nigeria [47–49]. The high incidence of *E. coli* associated with UTI among the pregnant women may be attributed to poor genital hygiene practices [50].

Multiparity, gestational age, history of UTI and anatomic urinary tract abnormalities are reported to affect the frequency of bacteriuria during pregnancy [51, 52]. Pregnant women in their 3rd trimester recorded the highest incidence of UTI (49.13%), followed by those in 2nd trimester (43.25%). Finding are conformity with studies from Bangladesh, Iran Ethiopia, Yemen, and India [48, 53–55]. Although Chi square exact test revealed a statistical association of UTI and gestational age (*p *= 0.002), it is in contrast to studies from Nigeria [20, 43]. Furthermore, the prevalence of UTI was found to increase with parity in this study. This findings however are in contrast to Emiru et al. [51] and Nandy et al. [56] studies.

*Escherichia coli* isolates were highly resistant to ampicillin, and tetracycline. Findings are similar to earlier studies in Ghana [21, 57, 58]. The high levels of resistance can be attributed to abuse of these drugs over the years because the drugs are relatively cheap and easily accessible [21, 58]. A considerable number of the bacteria harboured the *iut*A (aerobactin acquisition), *pap*C and *iha* (adhesins), *fyu*A and *irp*2 (iron capture systems), and *tra*T genes [59, 60]. In contrast to Sáez-López et al. [41] study with pregnant women in Barcelona, the ExPEC isolates in this study showed high antimicrobial resistance as previously reported in some African countries [61, 62]. In addition, the ampicillin resistant ExPEC isolates containing *Bla*_TEM_ gene showed a greater number of VFs in comparison with tetracycline or gentamicin resistant isolates. Our findings however, are dissimilar to Ramos et al. [63] study with pregnant women in Sweden, Uganda, and Vietnam [63]. The differences in the studies may be due to varying geographical area, host physiological changes or susceptibility to *E. coli* isolates with pathogenic islands containing VFs [64].

### Conclusion

In conclusion, our results demonstrates that antibiotic resistant ExPEC associated with UTI in some Ghanaian pregnant women have virulence properties which enables them to adhere, invade and utilize the iron acquisition systems. Information from this study is useful to develop appropriate interventions to avoid maternal and/neonatal infections with asymptomatic pathogens during obstetric care.

## Limitations

The study focused on asymptomatic infection rather than symptomatic infection and included only few hospitals in Ghana, thereby not allowing extrapolation of our results to other regions.

## Additional file

**Additional file 1: S1 File.** Questionnaire for demographic data collection. **S2 File.** Guidelines for interpreting antimicrobial susceptibility results. **S3 File.** Primers used for PCR. **S4 File.** Socio-demographic characteristics and distribution of UTI.

## Abbreviations

VFs: virulence factors
UTI: urinary tract infections
*E. coli*: *Escherichia coli*
ExPEC: extraintestinal pathogenic *E. coli*
PCR: polymerase chain reactions
CLED: cysteine lactose electrolyte deficient
CLSI: Clinical and Laboratory Standard Institute
