# Correction to: *Escherichia coli* bacteriuria in pregnant women in Ghana: antibiotic resistance patterns and virulence factors

**DOI:** 10.1186/s13104-019-4057-y

**Published:** 2019-01-16

**Authors:** Akua Obeng Forson, Wilson Bright Tsidi, David Nana-Adjei, Marjorie Ntiwaa Quarchie, Noah Obeng-Nkrumah

**Affiliations:** Department of Medical Laboratory Science, School of Biomedical and Allied Health Sciences, College of Health Sciences, University of Ghana, Legon, Accra, Ghana

## Correction to: BMC Res Notes (2018) 11:901 10.1186/s13104-018-3989-y

Following publication of the original article [1], the authors reported that one of the authors’ names was spelled incorrectly. In this Correction the incorrect and correct author name are shown. The original publication of this article has been corrected.

Originally the author name was published as:

- Noah Obeng-Nkuramah.

The correct author name is:

- Noah Obeng-Nkrumah.
