# Supplementary material for: Escherichia coli bacteriuria in pregnant women in Ghana: antibiotic resistance patterns and virulence factors
Source: BMC Res Notes. 2018 Dec 17;11:901. doi: 10.1186/s13104-018-3989-y (PMC6296117; doi:10.1186/s13104-018-3989-y)
Supplement: Supplementary file 1 — Additional file 1: S1 File. Questionnaire for demographic data collection. S2 File. Guidelines for interpreting antimicrobial susceptibility results. S3 File. Primers used for PCR. S4 File. Socio-demographic characteristics and distribution of UTI. [file 13104_2018_3989_MOESM1_ESM.docx]

**S1 File.** Questionnaire For Demographic Data Collection

1. Age of participant ( )

2. Age of the pregnancy (weeks/months)………………………………………………….

3. Is this your first pregnancy? ( ) Yes ( ) No

4. How many times have you given birth………………………………………………….

8. Marital status ( ) married ( ) single

9. Do you live with your partner? ( ) Yes ( ) No

10. Highest educational level

( ) Basic school level ( ) Second cycle level ( ) Tertiary level

11. Profession …………………………………………………………………………..

**S2 File.** Guidelines for interpreting antimicrobial susceptibility results

|  | Zone diameter (mm) | |
| --- | --- | --- |
| μg | Susceptible | Resistant |
| Ampicillin (10) | ≥17 | ≤13 |
| Tetracycline (30) | ≥15 | ≤11 |
| Cotrimoxazole (25) | ≥16 | ≤10 |
| Nalidixic acid (30) | ≥19 | ≤13 |
| Nitrofurantoin (300) | ≥17 | ≤14 |
| Gentamycin (10) | ≥15 | ≤12 |
| Cefuroxime (30) | ≥23 | ≤14 |

**S3 File.** Primers used for PCR.

|  |  | | **Primer Sequence (5’-3’)** | | | | | Amplicon | | | Annealing | | | |  |
| --- | --- | --- | --- | --- | --- | --- | --- | --- | --- | --- | --- | --- | --- | --- | --- |
| Category | **Primer** | Forward | | | | Reverse | **Size (bp)** | | Temp. (^o^C) | | | | Reference | | |
| Antibiotic resistance genes | *Bla_T_*_EM_ | gagtattcaacattttcgt | | | accaatgcttaatcagtga | | 857 | | | 64 | | [32] | |  |  |
|  | *Int* 1 | gggtcaaggatctggatttcg | | | acatgggtgtaaatcatcgtc | | 483 | | | 64 | | [33] | |  |  |
|  | *Int* 2 | cacggatatgcgacaaaaaggt | | | gtagcaaacgagtgacgaaatg | | 788 | | | 64 | | [33] | |  |  |
|  | *Aph(3’)-Ia*(aphA1) | atgggctcgcgataatgtc | | | ctcaccgaggcagttccat | | 600 | | | 64 | | [32] | |  |  |
|  | *Aph(3’)-Ia*(aphA2) | gaacaagatggattgcacgc | | | gctcttcagcaatatcacgg | | 680 | | | 64 | | [32] | |  |  |
| Adhesins | *pap*A | atggcagtggtgttttggtg | | | cgtcccaccatacgtgctcttc | | 717 | | | 64 | | [6] | |  |  |
|  | *pap*C | gtggcagtatgagtaatgaccgtta | | atatcctttctgcagggatgcaata | | | 200 | | | 64 | | [6] | |  |  |
|  | *pap*G I | ctgtaattacggaagtgatttctg | | | tccagaaatagctcatgtaacccg | | 1190 | | | 64 | | [6] | |  |  |
|  | *pap*GII, III | ctgtaattacggaagtgatttctg | | | actatccggctccggataaaccat | | 1070 | | | 64 | | [6] | |  |  |
|  | *sfa*/*foc* | ctccggagaactgggtgcatcttac | | | cggaggagtaattacaaacctggca | | 410 | | | 64 | | [6] | |  |  |
|  | *iha* | ctggcggaggctc tgagatca | | | tccttaagctc ccgcggctga | | 827 | | | 64 | | [34] | |  |  |
|  | *hra* | cagaaaacaaccggtatcag | | | accaagcatgatgtcatgac | | 260 | | | 64 | | [37] | |  |  |
|  | *ibe*A | aggcaggtgtgcgccgcgtac | | | tggtgctccggcaaaccatgc | | 171 | | | 64 | | [6] | |  |  |
| Toxins | *hly*D | ctccggtacgtgaaaaggac | | | gccctgattactgaagcctg | | 904 | | | 64 | | * | |  |  |
|  | *cnf1* | aagatggagtttcctatgcaggag | | | cattcagagtcctgccctcattatt | | 498 | | | 64 | | [6] | |  |  |
|  | *sat* | gcagctaccgcaataggaggt | | | cattcagagtaccggggccta | | 937 | | | 64 | | [35] | |  |  |
| Iron capture systems | fyuA | tgattaaccccgcgacgggaa | | | cgcagtaggcacgatgttgta | | 880 | | | 64 | | [6] | |  |  |
|  | *irp*2 | aaggattcgctgttaccggac | | | tcgtcgggcagcgtttcttct | | 287 | | | 64 | | [38] | |  |  |
|  | *iron* | aagtcaaagcaggggttgcccg | | | gacgccgacattaag acgcag | | 665 | | | 64 | | [34] | |  |  |
|  | *iu*C | cgccgtggctggggtaag | | | cagccggttcaccaagtatcactg | | 541 | | | 64 | | [36] | |  |  |
|  | *ire*A | gatgactcagccacgggtaa | | | ccaggactcacctcacgaat | | 254 | | | 64 | | * | |  |  |
| Protectins | *kpsM*TII | gcgcatttgctgatactgttg | | | catccagacgataagcatgagca | | 272 | | | 64 | | [6] | |  |  |
|  | *kpsMT*III | tcctcttgctactattccccct | | | tcctcttgctactattccccct | | 392 | | | 64 | | [6] | |  |  |
|  | *Omp*T | atctagccgaagaaggaggc | | | cccgggtcatagtgttcatc | | 559 | | | 64 | | * | |  |  |
|  | *tra*T | ggtgtggtgcgatgagcacag | | | cacggttcagccatccctgag | | 290 | | | 64 | | [6] | |  |  |
| Uropathogenic-specific protein | *usp* | acattcacggcaagcctcag | | | agcgagttcctggtgaaagc | | 440 | | | 64 | | [37] | |  |  |
| Aerobactin system | *iut*A | ggctggacatcatgggaactgg | | | cgtcgggaacgggtagaatcg | | 300 | | | 64 | | [6] | |  |  |

*J. R. Johnson protocols, Minneapolis VA Medical Centre, MN, US

**S4 File.** Socio-demographic characteristics and distribution of UTI

| Characteristics |  | Culture Results | |  | | |
| --- | --- | --- | --- | --- | --- | --- |
|  | Women  Tested (%) | No of  Significant growth (%) | No. of UTI associated. with  *E. coli* (%*)* | | χ^2^ | p value |
| **AGE (years)** |  |  |  | |  |  |
| 13 - 19 | 54 (13.5) | 24 (44.4) | 8 (33.3) | | 1.397 | 0.706 |
| 20 - 29 | 228 (57.0) | 96 (42.1) | 50 (52.1) | |  |  |
| 30 - 39 | 102 (25.5) | 42 (41.2) | 21 (50.0) | |  |  |
| 40 - 49 | 16 (4.0) | 9 (56.2) | 3 (33.3) | |  |  |
| **GESTATIONAL AGE** |  |  |  | |  |  |
| 1^st^ Trimester | 71 (17.8) | 20 (28.2) | 11 (55.0) | | 12.209 | 0.002* |
| 2^nd^ Trimester | 156 (39.0) | 66 (42.3) | 35 (53.0) | |  |  |
| 3^rd^ Trimester | 173 (43.3) | 85 (49.1) | 36 (42.4) | |  |  |
| **PARITY** |  |  |  | |  |  |
| Nulliparous | 129 (32.3) | 52 (40.3) | 30 (57.7) | | 1.025 | 0.599 |
| Primiparous | 107 (26.8) | 44 (41.1) | 18 (40.9) | |  |  |
| Multiparous | 164 (41) | 75 (45.7) | 34 (45.3) | |  |  |
| **EDUCATION** |  |  |  | |  |  |
| Basic Level | 251 (62.7) | 110 (43.8) | 50 (45.5) | | 2.678 | 0.262 |
| Secondary | 102 (25.5) | 46 (45.1) | 24 (52.2) | |  |  |
| Tertiary | 47 (11.75) | 15 (31.9) | 8 (53.3) | |  |  |
| **Hospitals** |  |  |  | |  |  |
| St Joseph Hospital | 80 (20.0) | 42 (52.5) | 23 (54.76) | | 9.847 |  |
| Volta Regional Hospital | 80 (20.0) | 28 (35.0) | 11 (39.29) | |  |  |
| Mary Theresa Catholic Hospital | 80 (20.0) | 39 (48.7) | 16 (41.03) | |  | 0.043* |
| Ketu South Municipal Hospital | 80 (20.0) | 36 (45.0) | 18 (50.00) | |  |  |
| St Anthony Hospital, Dzodze | 80 (20.0) | 26 (32.5) | 14 (53.84) | |  |  |
| **Total** | **400 (100.0)** | **171 (42.8)** | **82 (47.95)** | |  |  |

**Significant at* <0.005
